# Landscape of Prophylactic Strategies Against Human Parainfluenza Virus Type 3

**DOI:** 10.1002/rmv.70071

**Published:** 2025-10-09

**Authors:** Clémence Vacher, Julia Dubois, Marie‐Eve Hamelin, Guy Boivin, Manuel Rosa‐Calatrava

**Affiliations:** ^1^ CIRI Centre International de Recherche en Infectiologie Team VirPath Inserm Université Claude Bernard Lyon 1 CNRS, ENS de Lyon Lyon France; ^2^ Centre de Recherche en Infectiologie of the Centre Hospitalier Universitaire de Québec and Université Laval Québec QC Canada; ^3^ International Research Laboratory RESPIVIR France ‐ Canada, Centre Hospitalier Universitaire de Québec‐Université Laval Québec QC Canada; ^4^ Centre International de Recherche en Infectiologie Faculté de Médecine RTH Laennec Université Claude Bernard Lyon 1 Université de Lyon INSERM CNRS, ENS de Lyon Lyon France; ^5^ Virnext Faculté de Médecine RTH Laennec Université Claude Bernard Lyon 1 Lyon France

**Keywords:** monoclonal antibodies, parainfluenza virus type 3, respiratory infections, vaccine candidates

## Abstract

Human parainfluenza virus‐type 3 (HPIV3) is a respiratory virus responsible for mild illnesses in most of the population and severe illnesses, such as bronchiolitis and pneumonia, in young children, immunocompromised individuals and the elderly. To date, no vaccines nor antiviral drugs have been approved against HPIV3, despite its significant burden in vulnerable people. In this review, we present both past and current prophylactic strategies against HPIV3, such as live‐attenuated virus, vector‐based, subunit and mRNA vaccine candidates or antibody‐based passive protection. For each strategy, we give an overview of the most promising candidates evaluated in preclinical studies and we report immunogenicity and protection data from clinical trials. Finally, we discuss the most important challenges regarding those vaccine strategies and their potential administration to the different vulnerable populations.

## 1 Introduction

Human parainfluenza viruses (HPIVs) are enveloped single‐stranded negative RNA viruses belonging to the *Paramyxoviridae* family. Four antigenically distinct serotypes of HPIVs have been described (HPIV1 to HPIV4), with HPIV1 and HPIV3 belonging to the *Respirovirus* genus, and HPIV2 and HPIV4 to the *Rubulavirus* genus [1]. HPIVs are responsible for acute upper (URTI) and lower respiratory tract illnesses (LRTI) including croup, bronchiolitis and pneumonia in young children, immunocompromised and older adults [2, 3]. HPIVs circulate actively throughout the year, and human seropositivity reaches more than 90% by the age of 5, with cases of reinfection occurring throughout life [4, 5]. According to worldwide estimates, HPIVs were responsible for approximately 19 million LRTI cases among children younger than 5 years, including up to 1,26 million hospital admissions and 73,800 deaths in 2018 [4]. HPIVs are estimated to be the second or third leading cause of LRTI in the pediatric population after respiratory syncytial virus (RSV) and human metapneumovirus (HMPV), representing a substantial economic burden [5, 6]. Among the four HPIV serotypes, HPIV3 is the most frequent serotype that can be isolated throughout the year and associated with the most severe respiratory complications such as pneumonia [3, 7].

HPIV3 initiates infection of the respiratory tract by attachment to epithelial cells followed by fusion of virus and host cell membranes through the action of two viral glycoproteins: the hemagglutinin‐neuraminidase (HN) and the fusion (F) protein. HN binds to sialic acid residues at the surface of the host cell and triggers the activation of the F protein, promoting the membrane fusion and release of viral genome into the target cells [8]. Fusion is a dynamic phenomenon that results from conformational changes of the F protein from a metastable pre‐fusion (preF) to a post‐fusion (postF) state [8]. As surface glycoproteins, HN and F are the main viral antigens eliciting a neutralising antibody (NAb) response with scarce cross‐neutralisation between different HPIV serotypes [9, 10]. NAbs are likely to block the hemagglutinin activity [11] or the structural transition of the F protein, which results in altering cell attachment and fusion mechanisms. Three antigenic sites of HPIV3 preF have been characterised out of which two are located at the apex (site Ø and a site in DIII domain, [12, 13, 14]) and one at the side of the globular head (site X in DIII domain, [9]) of the glycoprotein.

Significant efforts have been made to develop vaccine strategies against HPIVs over the years, starting with formalin‐inactivated vaccine candidates (FI‐HPIV) in the 1960s. Unfortunately, these first candidates failed to protect infants and induced a lower NAb response than that elicited by natural infection [13, 15, 16]. Concomitantly, an RSV formalin‐inactivated vaccine candidate, belonging to the close *Pneumoviridae* family, accounted for the death of two immunised children who developed an enhanced respiratory disease (ERD) following natural RSV infection [17]. This event also hampered the development of HPIV vaccines for more than 4 decades. Following the failure of FI‐vaccines and based on new understanding of the immune response against HPIV3, vaccine development has focused on alternative vaccine platforms, such as live‐attenuated, vector‐based, subunit, and mRNA vaccines to elicit antibody responses against HN and/or F antigens. Strategies have focused on the response to the preF form since, like preF RSV, this conformation was proven to be more immunogenic than the postF [18]. Prophylactic antibodies have also been used for passive immunisation as a preventive approach against HPIV3 infections. Several HPIV3 vaccine candidates, combined or not with other respiratory viruses, showed promising results in preclinical studies (Tables 1 and 2) or clinical trials (Tables 3 and 4) and are discussed below.

**TABLE 1:** In vivo preclinical evaluation of live‐attenuated and vector‐based replicative vaccine candidates to prevent HPIV3 and other respiratory virus infections.

Category	Name	Description	Animal model (route of administration)	Dose	Attenuation^a^ (level)		NAb titers^d^ (targeted virus)	Viral titers after challenge (mean log_10_TCID_50_ or PFU/g)		Ref
					URT	LRT	25–35 days post‐immunisation	URT	LRT	
Live attenuated	cp45	Clinical strain HPIV3 JS attenuated through 45 cold passages	Chimpanzee (IT)	10^4^ TCID_50_	Yes (high)	Yes (high)	5.0–7.0	< LOD	0.5–1.7	[19]
			RM (IT)	10^5^ TCID_50_	Yes (high)	Yes (high)	9.7	2.1	2.3	[20]
				10^6^ TCID_50_	Yes (high)	Yes (high)	10.2	< LOD	< LOD	
	rcp45 (MEDI‐560)	Recombinant cp45 attenuated through the introduction of 15 mutations	Hamster (IN)	10^6^ PFU	Yes	Yes (high)	7.1	10^3^ to 10^4^‐fold reduction	10^3^ to 10^4^‐fold reduction	[21]
	BPIV3	Whole virus of the bovine parainfluenza type 3 strain	SM (IT)	10^7^ TCID_50_	Yes	Yes	5.1 (HPIV3)/7.6 (BPIV3)	1.1	1.1	[22]
			Chimpanzee (IT)	10^4^ TCID_50_	Yes	Yes (high)	9.5 (HPIV3)/7.5 (BPIV3)	—	—	
			OWL (IT)	10^7^ TCID_50_	Yes	Yes	—	—	—	
			RM (IT)	10^5^ TCID_50_ 2 × 10^5^ TCID_50_	Yes (high) Yes	Yes (high) Yes	— 3.7–5.1 (HPIV3)/5.6–7.8 (BPIV3)	— 0–0.5	— 0.3–1.0	[23] [22]
	HPIV3‐CPD	Min‐P, Min‐M and Min‐PM, CPD in the P and M genes individually or in combination	Hamster (IN)	10^5^ TCID_50_	Yes	Yes	8.8–10.5	< LOD	< LOD	[24]
		Min‐PFHN, Min‐MFHN, and Min‐PMFHN, CPD in the P, F, HN, and M genes individually or in combination			Yes	Yes	8.4–9.8	< LOD	< LOD	
Vector‐based replicative	rHPIV3‐F_B_ HN_B_ (rH/BPIV3)	HPIV3 vector with HN and F genes replaced by BPIV3 counterpart	RM (IT)	10^5^ TCID_50_ 10^5^ TCID_50_	Yes	Yes	4.7 (HPIV3) 4.5 (HPIV3)/9.5 (BPIV3)	2.6 3.0	1.8 1.0	[25] [26]
	rBPIV3‐F_H_ HN_H_ (rB/HPIV3)	BPIV3 vector with HN and F genes replaced by HPIV3 counterpart	RM (IT)	10^5^ TCID_50_ 2 × 10^5^ TCID_50_	Yes	Yes	6.8 (HPIV3)/3.8 (BPIV3) 5.7 (HPIV3)/2.7 (BPIV3)	2.5 < LOD	1.0 < LOD	[26]
										[23]
			Hamster (IN)	5 × 10^5^ PFU	Yes	Yes	128 (HPIV3)/6.7 (BPIV3)	< LOD	< LOD	[27]
	rHPIV3‐N_B_	HPIV3 vector with N gene replaced by BPIV3 counterpart	RM (IT)	10^5^ TCID_50_	Yes	Yes	7.5 (HPIV3) 8.2 (HPIV3)/6.5 (BPIV3) 9.8 (HPIV3)	2.1 2.3 2.6	1.2 1.4 1.5	[25] [26] [28]
	rB/HPIV3‐RSV F b/h PIV3/RSV F2 (MEDI‐534)	Insertion of RSV F A2 gene in the N‐P gene junction of rBPIV3‐F_H_ HN_H_	Hamster	10^6^ PFU	No^b^	No^b^	6.7 (HPIV3)/6.9 (RSV)	< 1.2 (HPIV3)/< 1.3(RSV)	< 1 (HPIV3)/< 1.6 (RSV)	[29]
			AGM (IT)	2 × 10^5^ ‐3x10^5^ PFU	—	—	5.8 (HPIV3)/4.1 (RSV A)/4.6 (RSV B)	< 1.1	< 1.0	[30]
	rB/HPIV3‐pre‐F RSV	(DS) insertion of RSV preF gene in the N‐P gene junction of rBPIV3‐F_H_ HN_H_	Hamster (IN)	10^5^ TCID_50_	Yes^b^	Yes^b^	10.5 (HPIV3)/9.1 (RSV)	‐ (HPIV3)/3–3.5 (RSV)	‐ (HPIV3)/<LOD (RSV)	[31]
	rB/HPIV3‐pre‐F RSV	(B3TMCT/DS) insertion of preF‐TMCT RSV gene in the N‐P gene junction of rBPIV3‐F_H_ HN_H_	Hamster (IN)	10^5^ TCID_50_	Yes (high)^b^	Yes (high)^b^	8.9 (HPIV3)/9.0 (RSV)	‐ (HPIV3)/2.9 (RSV)	‐ (HPIV3)/<LOD (RSV)	[32]
			RM (IT)	10^6^ TCID_50_	Yes (high)^c^	Yes^c^	7–8 (HPIV3)/9–10 (RSV)	—	—	
	rB/HPIV3‐pre‐F RSV GS/DS‐Cav1/B3TMCT	Insertion of preF‐TMCT RSV gene in the N‐P gene junction of rBPIV3‐F_H_ HN_H_	Hamster (IN)	10^5^ TCID_50_	Yes^b^	Yes (high)^b^	7.6 (RSV)	< LOD (5 out of 6 animals)	< LOD	[33]
			RM (IT)	10^6^ TCID_50_	—	—	6–8 (RSV)	—	—	
	rHPIV3‐RSV pre F F‐H3TMCT/N‐P	Insertion of preF‐TMCT RSV gene before the N gene or in the N‐P gene junction of rHPIV3	Hamster (IN)	10^6^ TCID_50_	No	No	12–14 (HPIV3)/12–14 (RSV)	< LOD (RSV)/‐ (HPIV3)	< LOD (RSV)/‐ (HPIV3)	[34]
	B/HPIV3/S‐2P	Insertion of the Wuhan‐Hu‐1 SARS‐CoV‐2 variant preS gene of in the N‐P gene junction of rBPIV3‐F_H_HN_H_	Hamster (IN)	10^5^ PFU	Yes (delayed)	No	1.95 log_10_ (WA1/2020) 1.97 log_10_ (B.1.1.7/Alpha) 1.72 log_10_ (B.1.351/Beta) ‐ (HPIV3)	< LOD (WA1/2020) ‐ (HPIV3)	< LOD (WA1/2020) ‐ (HPIV3)	[35]
				10^4.5^ TCID_50_	Yes (delayed)	Yes (delayed)	1.8 log_10_ (WA1/2020) 1.6 log_10_ (B.1.1.7/Alpha) 1.2 log_10_ (B.1.351/Beta) 3.4 log_10_ (HPIV3)	< LOD (WA1/2020) < LOD (B.1.1.7/Alpha) 1.5 (B.1.351/Beta) ‐ (HPIV3)	2.3 (WA1/2020) < LOD (B.1.1.7/Alpha) < LOD (B.1.351/Beta) ‐ (HPIV3)	[36]
Vector‐based replicative	B/HPIV3/S‐6P	Insertion of the Wuhan‐1 SARS‐CoV‐2 variant stabilised S gene in the N‐P gene junction of rBPIV3‐F_H_HN_H_	Hamster (IN)	10^4.5^ TCID_50_	Yes (delayed)	Yes (delayed)	1.8 log_10_ (WA1/2020) 1.7 log_10_ (B.1.1.7/Alpha) 1.3 log_10_ (B.1.351/Beta) 3.3 log_10_ (HPIV3)	< LOD (WA1/2020) < LOD (B.1.1.7/Alpha) 2.5 (B.1.351/Beta) ‐ (HPIV3)	< LOD (WA1/2020) < LOD (B.1.1.7/Alpha) < LOD (B.1.351/Beta) ‐ (HPIV3)	[36]
				10^5^ PFU	Yes (delayed)	Yes (delayed)	2.25 log_10_ (WA1/2020) 2.01 log_10_ (B.2.617.2/Delta) 0.87 log_10_ (B.1.1.529/Omicron)	2.5 (WA1/2020) < LOD (B.2.617.2/Delta) 2.6 (B.1.1.529/Omicron) ‐ (HPIV3)	< LOD (WA1/2020) 1.6 (B.2.617.2/Delta) 1.7 (B.1.1.529/Omicron) ‐ (HPIV3)	[71]
			RM (IN/IT)	10^6.3^ TCID_50_	Yes (delayed)	No	2.7–3.5 log_10_ (WA1/2020) 3.0–3.5 log_10_ (B.1.1.7/alpha) 1.4–1.8 log_10_ (B.1.1.529/Omicron BA.1) ‐ (HPIV3)	< LOD (WA1/2020) ‐ (HPIV3)	< LOD (WA1/2020) ‐ (HPIV3)	[72]
	B/HPIV3/S‐Delta‐6P	Insertion of the B.1.617.2/Delta SARS‐CoV‐2 variant stabilised S gene in the N‐P gene junction of rBPIV3‐F_H_HN_H_	Hamster (IN)	10^5^ PFU	Yes (delayed)	Yes (delayed)	2.25 log_10_ (WA1/2020) 2.45 log_10_ (B.2.617.2/Delta) 0.93 log_10_ (B.1.1.529/Omicron)	2.7 (WA1/2020) 1.7 (B.2.617.2/Delta) 1.7 (B.1.1.529/Omicron) ‐ (HPIV3)	< LOD (WA1/2020) < LOD (B.2.617.2/Delta) 1.7 (B.1.1.529/Omicron) ‐ (HPIV3)	[71]
	B/HPIV3/S‐Omicron‐6P	Insertion of the B.1.1.529/Omicron SARS‐CoV‐2 variant stabilised S gene of in the N‐P gene junction of rBPIV3‐F_H_HN_H_	Hamster (IN)	10^5^ PFU	Yes (delayed)	Yes (delayed)	0.89 log_10_ (WA1/2020) 0.91 log_10_ (B.2.617.2/Delta) 2.47 log_10_ (B.1.1.529/Omicron)	4.1 (WA1/2020) 2.0 (B.2.617.2/Delta) 1.7 (B.1.1.529/Omicron) ‐ (HPIV3)	1.6 (WA1/2020) 2.1 (B.2.617.2/Delta) 2.2 (B.1.1.529/Omicron) ‐ (HPIV3)	[71]
	b/h PIV3/hMPV F2	Insertion of HMPV A1 F gene in rBPIV3‐F_H_HN_H_	Hamster (IN)	10^6^ PFU	No^b^	No^b^	6.3 (HPIV3)/7.4 (HMPV)	< 1.2 (HPIV3) < 0.9 (HMPV)	< 1.2 (HPIV3)/< 0.5 (HMPV)	[29]
			RM (IT)	10^5.3^ PFU	Yes	Yes	—	—	—	[70]
			AGM (IT)	5 × 10^5^ PFU	—	—	7.2 (HPIV3)/7.1 (HMPV A)/2.7 (HMPV B)	‐ (HPIV3) 2.3 (HMPV A) ‐ (HMPV‐B)	‐ (HPIV3) < LOD (HMPV A) ‐ (HMPV‐B)	
	rPIV3(HA N‐P) rPIV3(HA P‐M) rPIV3(HA HN‐L)	Insertion of MV HA gene in the N‐P, P‐M, or HN‐L gene junction of JS HPIV3	Hamster (IN)	10^6^ TCID_50_	Yes (N‐P) No (P‐M) No (HN‐L)	No (N‐P) No (P‐M) No (HN‐L)	9 (HPIV3)/14.8 (MV) 9 (HPIV3)/15.3 (MV) 9.4 (HPIV3)/6.1 (MV)	2.0 (P‐M HPIV3)/‐ (MV)	3,2 (P‐M HPIV3)/‐ (MV)	[79]
	cp45 L(HA N‐P) cp45 L(HA P‐M)	Insertion of the MV HA gene in the N‐P or P‐M gene junction of HPIV3 containing 3 mutations in the *L* gene present in the cp45 strain.	Hamster (IN)	10^6^ TCID_50_	Yes (high) (N‐P) Yes (high) (P‐M)	Yes (high) (N‐P) Yes (high) (P‐M)	9.2 (HPIV3)/12.8 (MV) 10.8 (HPIV3)/13.4 (MV)	2.6 (N‐P HPIV3) 3.4 (P‐M HPIV3) ‐ (MV)	3.4 (N‐P HPIV3) 2.9 (P‐M HPIV3) ‐ (MV)	
	rHPIV3‐N_B_ HA	Insertion of MV HA gene in the P‐M gene junction of rHPIV3‐N_B_	RM (IT)	10^5^ TCID_50_	Yes	Yes	7.0 (HPIV3)/9.2 (MV)	1.2 (HPIV3) ‐ (MV)	2.3 (HPIV3) ‐ (MV)	[80]
	rHA_N‐P_ rHA_P‐M_ rHA_HN‐L_	Insertion of MV HA gene in the N‐P, P‐M or HN‐L gene junction of HPIV3	Hamster (IN)	10^6^ TCID_50_	Yes (N‐P) No (P‐M) No (HN‐L)	No (N‐P) No (P‐M) No (HN‐L)	9.5 (HPIV3)/12.4 (MV) 8.7 (HPIV3)/11.8 (MV) 9.0 (HPIV3)/8.1 (MV)	—	—	[78]
	r1HN_N‐P_ r1HN_P‐M_	Insertion of HPIV1 HN gene in the N‐P or P‐M gene junction of rHPIV3	Hamster (IN)	10^6^ TCID_50_	Yes (N‐P) Yes (high) (P‐M)	Yes (high) (N‐P) Yes (high) (P‐M)	9.0 (N‐P HPIV3)/3.4 (N‐P HPIV1) 7.2 (P‐M HPIV3)/2.7 (P‐M HPIV1)	—	—	
	r2HN_N‐P_ r2HN_P‐M_	Insertion of HPIV2 HN gene in the N‐P or P‐M gene junction of rHPIV3	Hamster (IN)	10^6^ TCID_50_	Yes (N‐P) No (P‐M)	Yes (N‐P) Yes (P‐M)	9.8 (N‐P HPIV3)/9.3 (N‐P HPIV1) 7.2 (P‐M HPIV3)/8.3 (P‐M HPIV1)	—	—	
	r1HN2HN	Insertion of HPIV1 HN gene in the N‐P and HPIV2 HN gene in the P‐M gene junction of rHPIV3	Hamster (IN)	10^6^ TCID_50_	Yes	Yes	9.0–9.6 (HPIV3) 3.8–4.8 (HPIV1) 8.3–10.5 (HPIV2)	2.6–3.9 (HPIV1) 3.0–3.6 (HPIV2) 1.6–1.9 (HPIV3)	2.1 (HPIV1) < 1.5–1.9 (HPIV2) < 1.5–1.7 (HPIV3)	
	rSeV‐hPIV3‐HN	Insertion of HPIV3 HN gene in the HN‐F junction of SeV	Cotton rat (IN)	2 × 10^6^ PFU	—	—	10	< LOD	< LOD	[76]
	rSeV‐hPIV3‐F	Insertion of HPIV3 F gene in the HN‐F junction of SeV	Cotton rat (IN)	2 × 10^6^ PFU	—	—	8	< LOD	< LOD	
	NDV‐LS/HN NDV‐BC/HN	Insertion of HPIV3 HN gene in the P‐M junction of NDV LS or BC	AGM (IT)	10^6.5^ PFU	— —	Yes (high) —	10.5 (HPIV3)/5.8 (NDV) 11.1 (HPIV3)/6.8 (NDV)	— —	— —	[77]
			RM (IT)		—	—	11.7 (HPIV3)/6.5 (NDV)	—	—	

Abbreviations: ‐: no information; AGM: African green monkey; IN: intranasal; IT: intratracheal; LOD: limit of detection; LRT: lower respiratory tract (lungs for rodents and trachea for NHP); NAb: neutralising antibody; NHP: non‐human primate; OWL: Old World monkey; RM: Rhesus monkey; SM: Squirrel monkey; URT: upper respiratory tract (nasal turbinate for rodents and nasopharynx for NHP).

^a^ Reduction in vaccine candidate titer compared to a reference virus depending on the study (HPIV3, rBPIV3‐F_H_ HN_H_^b^ or rB/HPIV3‐RSV‐F non‐optimised^c^), high attenuation is defined as a reduction fold ≥ 1000 compared to the reference virus.

^d^ NAb titers expressed in reciprocal mean log_2_ PRNT60 (60% plaque reduction neutralisation titer) or HAI (hemagglutination inhibition assay). Readout 28 days after the second vaccination.

**TABLE 2:** Preclinical evaluation of subunit vaccine candidates and prophylactic antibodies to prevent HPIV3 and other HPIV infections in animal models.

Category	Name	Description	Animal model	Dose	NAb titers^a^ (targeted virus)	Viral titers after challenge in mean log_10_ TCID_50_/g or PFU/g		Ref
						URT	LRT	
Subunit	OnlyEcto	Stabilisation in preF of HPIV3 F protein	Mouse	15 µg	8–10^b^	—	—	[37]
			Cotton rat	15 µg	—	2.0–3.0	< LOD	
	preF PIV1‐4	Stabilisation in preF of HPIV1, HPIV2, HPIV3 and HPIV4 F proteins	Mouse RM	2.5 µg 100 µg	11.5 (HPIV1)^c^/11.2 (HPIV2)^c^ 15.1 (HPIV3)^c^/12.1 (HPIV4)^c^ 11.2 (HPIV1)^d^/11.2 (HPIV2)^d^ 13.5 (HPIV3)^d^/10.9 (HPIV4)^d^	—	—	[18]
Prophylactic antibodies	PI3‐E12	Human monoclonal antibody targeting HPIV3 preF	Cotton rat	2.5–5 mg/kg	NA	< LOD	< LOD	[12]
	3x1	Human monoclonal antibody targeting HPIV3 preF and HPIV1 F protein	Hamster	5 mg/kg	NA	5–6 (HPIV3) 4 (HPIV1)	< LOD	[9]
	rPIV18	Human monoclonal antibody targeting HPIV3 preF	Cotton rat	2 mg/kg	NA	—	< 3	[11]
	rPIV3‐23 rPIV3‐28	Human monoclonal antibody targeting HPIV3 HN protein	Cotton rat	2 mg/kg	NA	—	< 3	
	4C03 4C06 1D10 1H09	Camelid heavy‐chain only antibodies targeting HPIV3 preF	Cotton rat	15 mg/kg	NA	< LOD < LOD 2.8 2.4	< LOD	[38]

Abbreviations: ‐: not done; LOD: limit of detection; LRT: lower respiratory tract (lungs for rodents and trachea for NHP); NA: not applicable; NAb: neutralising antibody; RM: Rhesus monkey; URT: upper respiratory tract (nasal turbinate for rodents and nasopharynx for NHP).

^a^ NAb titers expressed in reciprocal mean log_2_ PRNT60 or HAI.

^b^ Readout 15 days post‐immunisation.

^c^ Readout 35 days post‐immunisation^.^

^d^ Readout 126 days post‐immunisation.

**TABLE 3:** Former clinical trials of HPIV3 live attenuated vaccine candidates and vector‐based replicative competent vaccine candidates administered intranasally to prevent infections in the pediatric population.

Category	Name	Study	Participants (age, effective, sero status)	Vaccination scheme (dose, number, and interval if applicable)	Infection rate (%)	Serologic response* (% of responders)	IgA serologic response (% of responders*)	Main outcome	Ref
Live attenuated	cp45	Phase 1 Safety	6 months‐10 yo (41 SP 29 SN)	10^5^‐10^6^ TCID_50_ 10^2^‐10^5^ TCID_50_	11–28 43–88	0–11 43–81	25–22 25–38	Safe, immunogenic in SN children, and phenotypically stable.	[39]
		Phase 1 Safety and immunogenicity	18–40 yo and 15–59 months (36 SP) 6–36 months (31 SN) 1–2 months (33 UNS)	10^6^ PFU 10^4^–10^5^ PFU 10^4^ PFU (2, 1 or 3 months)	10–12 54–100 87–100	0 60–90 0–31	n.d 82–95 54–66	Well‐tolerated in all age groups. Immunogenic in SN children and infants (IgA response).	[40]
		Phase 1 transmission	6–48 months (24 SN)	10^5^ PFU	67	56	44	Transmission study, no transmission.	[41]
		Phase 2 Safety and immunogenicity	6–18 months (114 SN)	10^5^ PFU	n.d	79		Immunogenic and no occurrence of otitis media.	[42]
	rcp45 (MEDI‐560)	Phase 1 Safety and immunogenicity NCT00308412	6–36 months (5 SP,24 SN)	10^5^ TCID_50_ (2, 4–10 weeks)	40 92	20 88	—	Well‐tolerated and highly infectious in HPIV3 SN children. More potent and durable local immune response in older SN infants and children.	[43]
		Phase 1 Safety and immunogenicity NCT01254175	6–36 months (27 SN)	10^5^ TCID_50_ (2, 6 months)	96–100	88	—	Well‐tolerated and immunogenic in SN young children after 2 doses.	[44]
		Phase 1/2a Safety and immunogenicity NCT00508651	6‐< 12 months (20 SN)	10^5^ TCID_50_ (2 or 3, 7–9 weeks)	85	61.1–79.9	—	Infectious and immunogenic after 3 doses. Consistent with safety data previously reported for the biologically derived HPIV3cp45 vaccine.	[45]
	BPIV3	Phase 1 Safety	18–40 yo (18 SP)	10^6^‐10^7^ TCID_50_	44–56	0–44	0	Poorly infectious and immunogenic in adults.	[46]
		Phase 1 Safety and immunogenicity	6–60 months (24 SP, 31 SN)	10^5^‐10^6^ TCID_50_ 10^3^‐10^5^ TCID_50_	25–50 80–95	8 (HPIV3) 8 (BPIV3) 53–86 (HPIV3) 40–86 (BPIV3)	17–27 (BPIV3) 20–67 (BPIV3)	Infectious and immunogenic.	[47]
		Phase 1 Safety and immunogenicity	2–5.9 months (12 UNS) 6–36 months (11 SN)	10^5^ TCID_50_	92 89	42 (HPIV3) 67 (BPIV3) 70 (HPIV3) 85 (BPIV3)	—	Safe and infectious in infants younger than 6 months of age. Immunogenic in the majority of these young infants.	[48]
		Phase 2 Safety and immunogenicity	2 months (95 UNS)	10^5^‐10^6^ TCID_50_ (3, 2 months)	79	21–25 (HPIV3) 57–67 (BPIV3)	—	Higher incidence of low‐grade fever after dose 2. 3 doses likely provide sufficient immunity against HPIV3.	[49]
Vector‐based replicative	rBPIV3‐F_H_ HN_H_ (rB/HPIV3)	Phase 1 Safety and immunogenicity NCT00366782	18–49 yo and 15–59 months (25 SP) 6–36 months (14 SN)	10^6^ TCID_50_ 10^5^ TCID_50_	20 100	0–10 93	—	Both vaccine candidates were immunogenic in SN children with a higher proportion of vaccinees developing a serologic response when immunised with rBPIV3‐FH HNH_._	[50]
	rHPIV3‐N_B_	Phase 1 Safety and immunogenicity	18–49 yo and 15–59 months (25 SP) 15–59 months (14 SN) 6–36 months (7 SN)	10^5^ TCID_50_ 10^4^ TCID_50_ 10^5^ TCID_50_	0–10 71 86	0 64 86	—		[50]
	rB/HPIV3‐RSV F (MEDI‐534)	Phase 1 Safety NCT00111878	18–40 yo (61 SP)	10^4^‐10^6^ TCID_50_	0–5	0–5 (HPIV3) 0 (RSV)	—	MEDI‐534 was highly restricted in replication and did not boost RSV and PIV3 antibody titers in seropositive adults.	[51]
		Phase 1 Safety NCT00345670	1–9 yo (60 SP)	10^4^‐10^6^ TCID_50_	—	0–10 (HPIV3) 0–10 (RSV)	—	MEDI‐534 was minimally immunogenic in this SP pediatric population.	[52]
		Phase 1 Safety and immunogenicity NCT00493285	6‐< 24 months (62‐65 SN)	10^4^‐10^6^ TCID_50_	46.2–70	60.0–100 (HPIV3) 20.0–50.0 (RSV)	—	MEDI‐534 was poorly immunogenic for RSV due to mutations affecting RSV F protein expression.	[53, 54]
		Phase 1/2a Safety and immunogenicity NCT00686075	2 months 6‐< 24 months 1338 SN	10^4^‐ 10^6^ TCID_50_ (3, 2 months) 10^5^ ‐10^6^ TCID_50_ (3, 2 months)	NA	NA	NA	Insufficient immunogenicity against RSV.	[55]

Abbreviations: ‐: not done; d: day(s); mo: month(s); NA: not applicable; SP: seropositive; SN: seronegative; UNS: unscreened; yo: year(s) old.

* Serologic response was defined a ≥ 4‐fold increase from baseline in RSV titer by microneutralisation assay or HPIV3 and BPIV3 titer by HAI assay.

**TABLE 4:** Recent and ongoing clinical trials of HPIV3‐HMPV and HPIV3‐RSV‐HMPV vaccine candidates administrated intramuscularly (IM) or intranasally (IN).

Category	Name	Study	Description	Administration route	Participants (age, effective, sero status)	Vaccination scheme (dose, number, and interval if applicable)	NAb titer (GMFR)	Type of study and main outcome	Ref
mRNA	mRNA‐1653	Phase 1 Safety and dose selection NCT03392389	mRNA of HMPV and HPIV3 native F proteins	IM	18–49 yo (94 SP)	25–300 μg (1 or 2, 28 days)	6.04 (HMPV‐A) 6.33 (HMPV‐B) 3.24 (HPIV3)	Dose selection. Well‐tolerated, acceptable safety profile, and increase in HMPV and HPIV3 neutralising titers.	[56]
		Phase 1 Safety NCT04144348			12–55 months (17 SP)	10–30µg (2, 2 months)	2.9–6.1 (HMPV‐A) 6.2–13.2 (HMPV‐B) 2.8–3.0 (HPIV3)	Well‐tolerated, no safety concerns and a single injection boosted HMPV and HPIV3 antibody levels.	[57]
	mRNA RSV/HMPV/PIV3	Phase 1 Safety NCT06604767	mRNA of RSV, HMPV and HPIV3	IM	≥ 60 yo (390^a^)	NA	NA	Ongoing	[58]
		Phase 1 Safety NCT06850051			18–49 yo (270 SP^a^)	Low dose High dose			
Vector‐based replicative	B/HPIV3‐HMPV F‐B365 B/HPIV3‐HMPV‐PreF‐A	Phase 1 Safety NCT06546423	Insertion of HMPV A or B F gene in rBPIV3‐F_H_ HN_H_	IN	24–< 60 months (25 SP^a^)	10^5.6^ PFU	NA	Ongoing	

Abbreviations: d: day(s); mo: month(s); GMFR: geometric mean fold rise, defined as the geometric mean ratio of postbaseline/baseline titers; NA: not applicable; NAb: neutralising antibody; SP: seropositive; yo: year(s) old.

^a^ Total number of enrolled participants.

### 1.1 Live‐Attenuated Vaccines

Live‐Attenuated Vaccines (LAVs) are replication‐competent viruses modified to reduce their pathogenicity [14]. LAVs induce systemic and local immune responses by mimicking natural infection [59]. A major challenge for this approach is to balance viral attenuation and immunogenicity. First LAVs have been generated by spontaneous genome mutations induced by subsequent passages of viral strains in cells in conditions of suboptimal growth. One potential risk is the reversion of the mutations towards the wild‐type genome, leading to the reversal of attenuated phenotype thus restricting the use of LAVs in immunocompetent individuals. Reverse genetic systems have allowed the generation of customised recombinant viruses to reduce this reversion risk by introducing supplementary mutations, that is gene deletion, and/or codon pair deoptimisation [60]. Another LAV approach is to use animal counterpart viruses, such as bovine parainfluenza virus type 3 (BPIV3), that are naturally attenuated in humans due to host restriction [22].

#### 1.1.1 Cold‐Passaged Vaccine Candidate

Cp45 vaccine candidate is a cold‐adapted (*ca*) virus that was generated by 45 subsequent passages of the JS HPIV3 clinical strain on Vero cells at 20°C [61]. Cp45 was proven to be genetically stable and to retain a temperature sensitive phenotype (*ts*) above 35°C *in vitro*. When administered intranasally and intratracheally to non‐human primates (NHP), cp45 induced a strong hemagglutination‐inhibiting (HAI) antibody response, conserved its attenuation phenotype without causing any symptoms of rhinorrhea and protected NHP against HPIV3 challenge (Table 1) [19, 20].

Several clinical studies were conducted with cp45 (Table 3). Single intranasal (IN) administration of the vaccine candidate in seronegative children induced a 4‐fold increase in HAI titer in 81% of the vaccinated children [39]. However, the same and higher doses were poorly immunogenic in seropositive children because of pre‐existing immunity [39, 40]. Another Phase 1 study reported that cp45 administration was well tolerated in all age groups and induced a significant HAI antibody response in 60%–90% of vaccinated seronegative children but not in seropositive individuals [40]. The administration of a single dose of cp45 infected more than 87% of infants aged 1–2 months, but a HAI response was observed in less than 30% of them. This HAI response might be due to the interfering presence of maternal antibodies. Nonetheless, serum anti‐HN IgA were measured in 54%–66% of the infants, suggesting that IgA titers could be a good correlate of protection [40].

A subsequent Phase 1 study showed no transmissibility between vaccinated and unvaccinated children from South Africa. Interestingly, the antibody response was lower in African than the one observed in American children (56% vs 81%–95%, respectively) of the same age vaccinated with the same dose [41]. This difference remained undefined, highlighting the discrepancies that can exist in populations of different ethnicities. The Phase 1 study was followed by a Phase 2 clinical trial, where it was reported that one dose of 10^5^ PFU of cp45 induced an antibody response in 79% of seronegative children [42].

Rcp45 or MEDI‐560, a cp45 recombinant counterpart LAV, was generated to reduce contamination risks with adventitious agents and to control the number of passages [43]. The 15 amino acid substitutions conferring cp45 attenuated phenotype were identified, and reported to be distributed throughout the genome [21]. Thus, rcp45 was validated in preclinical models for its similarities with cp45 regarding its attenuation and phenotypic stability (Table 1) [21]. Three clinical studies (Table 3) confirmed that rcp45 was comparable to cp45 regarding tolerability and immunogenicity. In phases 1, one dose of rcp45 in seronegative children aged 6–36 months induced a significant serological response in 61.1%–88% of the vaccinees [43, 44, 45] (NCT00308412, NCT01254175, NCT00508651). The replication of rcp45 was restricted after a second IN administration still, an increase in antibody response was observed in 14%–23% of the vaccinees [43, 44]. In a Phase 1/2 study, a three dose vaccination regimen resulted in an overall antibody response in 76.9% of the vaccinated infants compared to 61.1% and 73.3% after one or two doses, respectively [45] (NCT00508651). Together, those results suggested that a 2 to 3‐dose rcp45 vaccination schedule with a 3–6 months interval between doses should be considered, since immunity to respiratory viruses wanes within this time interval [43, 44]. Despite these promising results, no supplementary Phase 2 or 3 studies were conducted with the rcp45 vaccine candidate.

#### 1.1.2 Codon Pair Deoptimisation

Codon pair deoptimisation (CPD) is an attenuation strategy consisting of replacing codons within viral genomes with codons considered suboptimal in the host cell. The use of suboptimal codons affects mRNA stability and/or structure as well as tRNA‐ribosome loading, which can reduce translation of viral proteins during the infection. This strategy presents several advantages in vaccine development. The amino acid sequence remains unchanged and the viral epitopes are identical to the parental strain. The higher frequency of CpG and UpA dinucleotides in the viral nucleic acid might trigger a stronger host innate immune response. Mutations are numerous, which renders reversion unlikely, and can be combined with other attenuation strategies [62].

Using CPD in one or several HPIV3 genes, Afroz et *al*. generated and evaluated 12 HPIV3‐CPD candidates [24]. Vaccine candidates bearing CPD in N and/or L genes were excessively attenuated in vitro and were not further characterised. Most of the other modified viruses, presented in Table 1, were significantly attenuated, especially in A549 cells. Of note, a stronger induction of the innate immune response, especially the IFN‐induced response, was observed in A549 cells compared to Vero cells infected with the HPIV3‐CPD candidates, except the ones with CPD in the M gene. Those HPIV3‐CPD candidates were all attenuated in hamsters, induced significant antibody titers (8.1–10.4 log_2_), and protected animals after challenge with HPIV3. Interestingly, *in viv*o expression of genes associated with the IFN pathway was higher after vaccination with vaccine candidates than after infection with a wild‐type HPIV3. Still, the magnitude of the innate immune response did not correlate with higher restriction of viral replication nor with higher content of CpG and UpA dinucleotides within viral genomes. Overall, HPIV3‐CPDs were described as promising vaccine candidates or as potential vectors to express foreign antigens [24].

#### 1.1.3 Host‐Restricted Vaccine Candidate

The genetic and antigenic similarities between bovine parainfluenza virus type 3 (BPIV3) and HPIV3 led to the use of BPIV3 as a host‐restricted vaccine candidate to protect against infection with HPIV3 [63]. BPIV3 was first tested in NHP and proved to be immunogenic and protective against HPIV3 infections (Table 1), encouraging its use in human clinical trials (Table 3) [22].

In a first clinical study in HPIV3 seropositive adults, an IN administration of BPIV3 was poorly immunogenic with an increase in HPIV3 NAbs in 0% or 44% of the participants depending on the dose [46]. Similar findings were observed in seropositive children where an immunisation with BPIV3 resulted in an increase in HPIV3 NAbs in only 8% of them [47]. However, BPIV3 was reported immunogenic in seronegative children aged 6–60 months with an increase in HPIV3 NAbs in 60%, 86%, and 53% of the children receiving corresponding escalating doses [47]. In a third Phase 1 study, infants aged 2 to < 6 months were less responsive than seronegative children, with NAbs response to HPIV3 observed in only 42% of the vaccinees, suggesting an interfering effect of maternal antibodies against HPIV3 [48]. In a Phase 2, unscreened 2‐month‐old infants received 3 doses of BPIV3 with a 2‐month interval, and only 21% and 25% of them had an increase in HPIV3 NAbs [49]. Given the limited immune response in vaccinees, BPIV3 was then used as a backbone for vector‐based vaccine candidates.

### 1.2 Vector‐Based Replicative Vaccine Candidates

Vector‐based replicative vaccines are recombinant viruses genetically modified to express one or more exogenous antigen(s) in infected cells [64]. Like LAVs, their efficacy is based on a good balance between low pathogenicity and immunogenicity, without the use of adjuvant. Viral vaccine vectors are usually host‐restricted and/or carry additional mutations and gene deletions. Different vector‐based replicative competent vaccine candidates were developed to protect against HPIV3 and other respiratory viruses such as RSV, HMPV, measles virus (MV) and SARS‐CoV‐2.

#### 1.2.1 BPIV3 Vector‐Based HPIV3 Vaccine Candidates

Several rBPIV3/HPIV3 recombinant viruses were developed by replacing HPIV3 genes with BPIV3 counterparts, and inversely, to increase immunogenicity towards HPIV3 [25]. Among them, rHPIV3 expressing BPIV3 N protein (rHPIV3‐N_B_) and rBPIV3 expressing HPIV3 HN and F glycoproteins (rBPIV3‐F_H_HN_H_) were described as potential vaccine candidates against HPIV3 because of their immunogenicity and protection induced in hamsters and rhesus monkey models [23, 26, 27, 28].

In a clinical trial in HPIV3 seropositive adults and children, rBPIV3‐F_H_HN_H_ and rHPIV3‐N_B_ were poorly immunogenic when administered intranasally [50] (NCT00366782). In contrast, both candidates were immunogenic in seronegative children who developed a serological response when immunised with rBPIV3‐F_H_HN_F_ (93%) or with rHPIV3‐N_B_ (86%). Despite these promising results, rBPIV3‐F_H_‐HN_H_ was discontinued, and a multivalent strategy using rBPIV3‐F_H_HN_F_ as a backbone was prioritised. This rBPIV3‐F_H_HN_H_ virus was engineered to also express the RSV F antigen [65]. As a bivalent vaccine candidate, rB/HPIV3‐RSVF or MEDI‐534 (MedImmune) was validated in various animal models for its immunogenic and protective properties (Table 1) [29, 30, 66, 67]. Similar to rBPIV3‐F_H_HN_H_, an IN administration of MEDI‐534 was poorly immunogenic in seropositive individuals (NCT00111878, NCT00345670, Table 3) [51, 52]. However, 3 administrations were immunogenic in seronegative children. Indeed, 80%, 60%, and 100% of the vaccinees developed NAbs against HPIV3, and 44.4%–55.6% of them developed a serological response against RSV (NCT00493285) [53]. Nasal wash samples from vaccinated children with confirmed MEDI‐534 shedding from this study were sequenced. The results showed that 55% of the 24 sequences contained a subpopulation with mutations, resulting in premature stop codons that certainly reduced RSV‐F expression and therefore immunogenicity toward RSV [54]. This genetic instability was confirmed in vitro following passages in human lung cell line MRC‐5 [68]. Another Phase 1 (NCT00686075) confirmed this poor serological response against RSV in a larger cohort of vaccinated children, showing that MEDI‐534 needed optimization to reach higher RSV immunogenicity [55].

In this context, Liang et *al*. aimed at increasing the stability and immunogenicity of the RSV F protein. Different RSV F gene insertion positions (pre‐N, N‐P, P‐M, and HN‐L) within the rBPIV3‐F_H_HN_H_ genome were tested. The pre‐N or N‐P positions resulted in higher expression and immunogenicity in hamsters, as previously observed with MEDI‐534 [29, 30, 66, 67, 69]. The RSV F gene was then codon‐optimised and stabilised in its preF conformation which increased its expression *in vitro* and its immunogenicity by 2.8 to 10.6‐fold compared to native RSV F in hamsters [31]. Finally, RSV preF transmembrane (TM) and cytoplasmic tail (CT) domains were replaced by those of BPIV3, significantly enhancing the packaging of the antigen into the viral particle. This in turn resulted in 100‐fold increase in immunogenicity in NHPs [32] and a protection of hamsters against RSV challenge [32, 33].

Analogous rBPIV3/HPIV3‐based vaccine candidate (b/hPIV3/hMPV F2) expressing a native HMPV F antigen was engineered and proved to be immunogenic and protective in hamsters [29]. As observed for MEDI‐534, NHPs vaccinated with b/hPIV3/hMPV F2 were protected against HMPV A challenge. The authors also evaluated the cellular (IFN‐γ secreting T cells) and humoral HMPV B specific responses. Despite lower NAbs titers, these results suggested that rB/HPIV3‐HMPV F could protect against infections with both HMPV subtypes [70]. An ongoing Phase 1 study (NCT06546423) in seropositive children aged 24–60 months, evaluates two bivalent vaccine candidates expressing HMPV A and B proteins (B/HPIV3/HMPV‐PreF‐A and B/HPIV3/HMPV‐F‐B365, respectively) and administered by nasal spray (Table 4).

The rBPIV3/HPIV3 platform was also used to express stabilised prefusion conformation of the spike (S) protein from several SARS‐CoV‐2 variants. The first vaccine candidate, B/HPIV3/S‐2P, composed of the Wuhan‐Hu‐1 S protein stabilised with 2 proline residues, was immunogenic against HPIV3 and three SARS‐CoV‐2 variants (WA1/2020, B.1.1.7/Alpha, B.1.351/Beta) [36], and protective against WA1/2020 variant challenge in hamsters [35, 36]. The S protein was further stabilised by the introduction of 4 additional proline residues. The resulting vaccine candidate B/HPIV3/S‐6P induced HPIV3 NAbs in hamsters and NAbs against WA1/2020, B.1.1.7/Alpha, B.1.351/Beta, B.2.617.2/Delta, and to a lesser extent against B.1.1.529/Omicron in hamsters and/or rhesus monkeys (Table 1) [36, 71, 72]. Finally, two additional vaccine candidates expressing the prefusion S of B.1.617.2/Delta or B.1.1.529/Omicron variant induced mucosal and serum anti‐S antibodies as well as NAbs, and protected animals against a homologous or heterologous challenge with WA1/2020, B.1.617.2/Delta or B.1.1.529/Omicron strains (Table 1). The authors suggested that those vaccine candidates could be potential pediatric vaccines to immunise against SARS‐CoV‐2 and HPIV3 [35, 36, 71, 72].

#### 1.2.2 SeV and NDV Vector‐Based HPIV3 Vaccine Candidates

Sendai virus (SeV), the murine *respirovirus*, is an interesting viral vector to develop vaccine candidates because of its proximity to HPIVs and its natural host range restriction in humans [73]. In clinical studies in adults and seropositive children, SeV was safe and induced an antibody response against HPIV1 [74, 75]. SeV was then modified to express either the HN or F protein of HPIV3 (rSeV‐hPIV3‐HN and rSeV‐hPIV3‐F) [76]. Both rSeV‐hPIV3‐HN and rSeV‐hPIV3‐F vaccine candidates induced humoral (NAb titers of 10 and 8log_2_, respectively) and cellular (IFN‐γ secreting T cells) responses against HPIV3 in cotton rats while protecting animals against HPIV3 challenge (Table 1). Interestingly, rSeV‐hPIV3‐HN was also co‐administered with another rSeV‐based vaccine candidate expressing RSV F protein and conferred protection against RSV, HPIV3 and HPIV1 following challenges with each wild‐type virus [76].

Another paramyxovirus, Newcastle disease virus (NDV), has been widely used for more than 20 years to design recombinant virus vaccine candidates expressing different viral antigens [60]. The lentigenic vaccine strain LaSota (NDV‐LS) and the mesogenic strain Beaudette C (NDV‐BC) were both modified to express the HN protein of HPIV3 (NDV‐LS/HN and NDV‐BC/HN). These candidates led to the induction of HAI titers of 10.5 and 11.4 log_2_ against HPIV3 in NHP (Table 1), suggesting an efficient protection against HPIV3 [77].

#### 1.2.3 HPIV3 Vector‐Based Vaccine Candidates

HPIV3 was also used as a vector and engineered to express the antigens of MV, HPIV1‐2, and RSV [78]. The integration of one foreign antigen was sufficient to confer attenuation and low pathogenicity to the recombinant HPIV3 viruses, while conserving an efficient replication in vitro [34, 78, 79, 80].

Several recombinant HPIV3‐MV candidates, detailed in Table 1, were designed to express the main antigen of MV, the hemagglutinin protein (HA). Different HPIV3 backbones were used: the HPIV3 clinical strain JS, the attenuated cp45 L virus, containing three mutations in the *L* gene identified in cp45, and the recombinant rHPIV3‐N_B_. The corresponding HPIV3 JS, cp45 L or rHPIV3‐N_B_ vaccine candidates induced a marked increase in NAb response against HPIV3 and MV in hamsters or in NHP [80].

Similarly, other HPIV3‐based constructs were developed and different position for the insertion of MV HA, HPIV1 and/or HPIV2 HN genes were evaluated [78]. Despite their attenuated profiles in the respiratory tract of hamsters, the MV (rHA_N‐P_, rHA_P‐M_ and rHA_HN‐L_), HPIV1 (r1HN_N‐P_ and r1HN_P‐M_) and HPIV2 (r2HN_N‐P_ and r2HN_P‐M_) vaccine candidates induced significant NAb titers against HPIV3 and MV, HPIV1 or HPIV2, respectively (Table 1). IN immunisation of hamster with the vaccine candidate r1HN2HN, expressing both HPIV1 and HPIV2 HN proteins, induced NAb response and protected animals against challenges with HPIV1, HPIV2 or HPIV3 [78].

The HPIV3 JS strain was also used to develop an RSV/HPIV3 vaccine candidate (F‐H3TMCT/N‐P) expressing the preF antigen [34]. The TM and CT domains of the preF were substituted by their HPIV3 counterparts to increase its packaging efficacy. Overall, FH3TMCT/N‐P was immunogenic and protective against RSV challenge in hamsters. Those different HPIV3 vector‐based vaccine candidates were not evaluated in clinical trials.

### 1.3 Subunit Vaccines

Subunit vaccines are purified recombinant whole or partial viral antigens that can elicit a protective immune response. Being replication‐deficient, subunit vaccines are considered safer for use in pregnant women, immunocompromised individuals and the elderly. However, they usually require the addition of adjuvant to stimulate efficient immunity [81]. While most commercialised subunit vaccines are administered via intramuscular (IM) injection, the IN route could be advantageous to stimulate mucosal immunity [82, 83, 84]. Currently, approaches to develop HPIV3 subunit vaccines are focusing on the use of preF protein. Stewart‐Jones et *al*. developed a quadrivalent vaccine candidate (preF PIV1‐4) targeting the four HPIV serotypes [18]. The vaccine consists of the four preF proteins stabilised through the introduction of disulfide bonds, cavity‐filling mutations, and the trimerisation of their C terminus domain with GCN4 motif. Immunisation of mice with poly(I:C) adjuvanted preF candidate led to higher NAb titers compared to mice immunised with the postF proteins of HPIV1‐4 with respectively 9.3‐, 17‐, 450‐, and 9.7‐fold increase in NAb titers for HPIV 1, 2, 3 and 4. This observation was confirmed in NHPs immunised with poly(I:C) adjuvanted preF candidate (Table 2) [18].

Another HPIV3 preF subunit candidate, OnlyEcto, was also developed with additional substitutions to stabilise the head domain and without the GCN4 motif to avoid off‐target responses [37]. An IM administration of this preF candidate was more immunogenic in naive mice than the postF with a 19.8‐fold increase in NAb titers (Table 2). This vaccine candidate significantly increased the immune response of mice pre‐exposed to HPIV3 infection, suggesting its efficacy, in seropositive individuals [37].

### 1.4 mRNA Vaccines

In response to the COVID‐19 pandemic, mRNA vaccine platforms were rapidly developed. The encapsulation of mRNA molecules in lipid nanoparticles (LNPs) enhanced the delivery of mRNA into the cytoplasm and the translation was improved by the addition of modified nucleotides [85]. Mainly IM‐administered, those vaccines do not require adjuvants since LNP carriers also act as such. An important inconvenience of this technology is the very low‐temperature storage (−80°C) required for vaccine stability, making worldwide distribution complex [85, 86].

Currently, HPIV3 mRNA vaccine candidates under evaluation (Table 4) are combined with other mRNA vaccine candidates to provide a broader protection against different respiratory viruses. For example, single or double administration of the mRNA‐1653 candidate (Moderna), encoding for the native F proteins of HPIV3 and HMPV, was evaluated in Phase 1 studies in seropositive adults and children (NCT03392389, NCT04144348) [56, 57]. The first dose induced a significant increase in NAb titers, which remained similar after a second dose, for each virus HMPV‐A, HMPV‐B and to a lesser extent, HPIV3. While both preF and postF were expected to be expressed, the antibody response was biased toward the preF conformation for all viruses, suggesting that a preF stabilisation could further improve the immunogenicity of the mRNA‐1653 vaccine candidate [56]. Overall, mRNA‐1653 was considered safe, well‐tolerated, and immunogenic in seropositive adults and children [57]. However, mRNA vaccines in younger children should be considered with precaution since clinical trials with two RSV‐F mRNA vaccine candidates in children < 2 years old were recently suspended by the FDA due to cases of vaccine‐enhanced disease [87, 88].

Another mRNA candidate developed by Sanofi Pasteur, a trivalent RSV/HMPV/PIV3 mRNA vaccine, is currently evaluated in a Phase 1 (NCT06604767) with participants aged > 60 years old [89]. Different doses and combinations of RSV/hMPV/PIV3, RSV/hMPV, or only RSV vaccines are also being tested in adults (NCT06850051).

### 1.5 Prophylactic Antibodies

Monoclonal antibodies (mAbs) targeting a specific epitope, preferably highly conserved within viral proteins playing a major role in viral replication, might be used as prophylaxis. Three mAbs commercialised to prevent RSV infection in infants, Palivizumab (Synagis, AstraZeneca) [90], Nirsevimab (Beyfortus, Sanofi) [91] and Clesrovimab‐cfor (Enflonsia, Merck) [92], target epitopes on the F glycoprotein and inhibit the fusion of membranes and viral entry [93, 94]. More efficient than Palivizumab, Nirsevimab targets the Ø antigenic site exposed in preF conformation [95]. Similarly, mAbs in development against HPIV3 (Table 2) also focus on targeting the more immunogenic preF sites [18].

PI3‐E12 is a human mAb targeting the antigenic site Ø, located on the apex of the HPIV3 preF [12]. Administration of PI3‐E12 in cotton rats prevented HPIV3 replication in lungs and induced a significant decrease of viral titers in nasal turbinates after the challenge. In immunocompromised cotton rats, the injection of PI3‐E12 after infection with HPIV3 decreased viral titers in the lungs by 28‐fold, suggesting that PI3‐E12 could be also used as a therapeutic agent. Nonetheless, further studies in NHP are necessary to better characterise such therapeutic potential [12].

The mAb 3x1 targets the X antigenic site located between the equator and the apex of HPIV3 preF and also binds to HPIV1 F protein, likely at a site located within the HRA helix [9]. The administration of mAb 3x1 in hamsters, followed by an infection with HPIV1 or HPIV3, significantly reduced viral replication of both viruses in the lungs but only limited the replication of HPIV1 in nasal turbinates. The co‐administration of 3x1 and MxR, a mAb targeting HMPV and RSV F proteins, two days before HPIV3 and RSV co‐infections, reduced HPIV3 and RSV pulmonary viral loads in hamsters by 88‐fold and 17‐fold, respectively. However, this mAb combination had a lower efficacy in nasal turbinates characterised by 2.9‐fold decrease of RSV replication and no impact on HPIV3 replication. It was suggested that combinations of several antibodies could be used to confer broader protection against respiratory viruses [9].

The HN protein is another major surface antigen of HPIV3 and Suryadevara et *al*. developed two mAbs binding to the HN (rPIV3‐23 and rPIV3‐28) in addition to rPIV‐18 binding to the preF apex in DIII domain. The authors demonstrated that rPIV3‐18 IC50 was higher than those of anti‐HN antibodies (138 ng/ml vs 21 and 40 ng/ml, respectively) indicating that mAbs targeting the HN protein could be a promising prophylactic strategy. When administered to cotton rats, each antibody significantly reduced lung viral titers [11].

More recently, four single‐chain camelid antibodies, 4C03, 4C06, 1D10, and 1H09, were characterised by Johnson et *al*. They bind with high affinity to HPIV3 preF by targeting other antigenic sites than those already identified (Ø, X, and rPIV18 epitope) [38]. The recognition domain of camelid antibodies (VHH) is only composed of two variable heavy chains, that can therefore target more efficiently antigenic sites less accessible to human or mouse antibodies. VHH domains were fused with Fc fragments to increase serum half‐life and activate immune complement‐based and cellular responses. In cotton rat model, the injection of the four modified VHHs prevented HPIV3 replication in lungs of infected animals, but only 4C03 and 4C06 also prevented replication in nasal turbinates.

## 2 Conclusion

**FIGURE 1:**
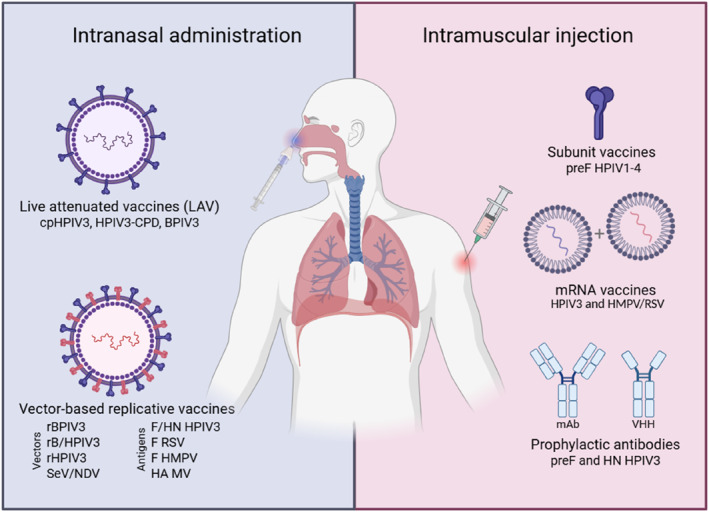
Illustration of the main prophylactic strategies, administered intranasally or intramuscularly, developed against HPIV3 and other respiratory viruses. BPIV3: bovine parainfluenza type 3; cp: cold‐passaged; CPD: codon pair deoptimisation; F: fusion protein; HA: hemagglutinin protein; HMPV: human metapneumovirus; HN: hemagglutinin neuraminidase protein; HPIV3: human parainfluenza type 3; HPIV1‐4: human parainfluenza virus type 1, 2, 3 and 4; mAb: monoclonal antibody; MV: measles virus; NDV: Newcastle disease virus; preF: pre‐fusion F protein; RSV: human respiratory syncytial virus; SeV: Sendai virus; VHH: recognition domain of camelid antibodies also known as nanobodies.

Over the years, HPIV3 vaccine development has led to the elaboration of numerous prophylactic strategies (Figure 1). Some of those reached clinical trials, however, no market‐approved vaccine is currently available. LAV or vector‐based replicative vaccines administered intranasally were demonstrated to be safe and well‐tolerated in the pediatric population. They mimic natural infection which stimulates both systemic and mucosal immunity. They also confer protection at the entry site of pathogens, which could reduce the virus transmission into the population. Two promising LAV candidates (MEDI‐560 and BPIV3) were first characterised, followed by the development of vector‐based replicative vaccine candidates providing broader protection against different human respiratory viruses, such as HMPV, RSV, HPIV1‐2, SARS‐CoV‐2 and MV. Of note, the rBPIV3‐F_H_HN_H_ backbone used to develop a bivalent HPIV3/RSV vaccine (MEDI‐534; NCT00686075, NCT00493285) has been recently evaluated in a Phase 1 clinical trial with two other bivalent vaccine candidates expressing HMPV A and B F proteins (rB/HPIV3‐HMPV‐F‐B365 and B/HPIV3‐HMPV‐PreF‐A, NCT06546423). Despite having numerous advantages, previous studies have shown that the recombinant genome can be unstable and needs optimization to reach optimal exogenous gene expression for immunisation and their use as a multivalent vaccine. Candidates generated by codon pair deoptimisation (HPIV3‐CPD) were recently evaluated in preclinical models with encouraging results, and might constitute a promising alternative.

The Covid‐19 pandemic has allowed rapid development of mRNA vaccine platforms. Indeed, two candidates targeting HPIV3, HMPV and/or RSV are currently evaluated in the elderly (mRNA RSV/HMPV/PIV3, NCT06604767) and in pediatric (mRNA‐1653, NCT04144348) populations. However, mRNA vaccines can rise safety concerns in young children [87]. Other approaches targeting the preF, similar to the ones marketed or in development against RSV, such as subunit vaccines (OnlyEcto and preF PIV1‐4) and prophylactic antibodies, were recently evaluated in preclinical models against HPIV3.

The age and serological status of the individuals should be considered to determine the appropriate vaccination approach for a given population [96]. Adjuvanted HPIV3 preF subunit vaccine candidates could be administered to the elderly, whereas LAV could be used to immunise infants older than 6 months. Finally, prophylactic antibodies could be passively administered in infants under 6 months and used as a therapeutic agent in immunocompromised individuals. To date, only LAV are delivered intranasally, but new strategies have been or are being developed for IN administration of mRNAs, subunit vaccines and mAbs, notably for respiratory viruses such as Influenza [97, 98], RSV [99] and SARS‐CoV‐2 [100, 101, 102, 103, 104]. These approaches could also be applied to new HPIV3 vaccines to stimulate mucosal immunity and induce durable protection, without excluding heterologous booster strategies.

## Author Contributions

Conceptualisation: J.D. and M.R.‐C. writing – original draft preparation: C.V., J.D. Writing – review and editing: J.D., M.R.‐C., M.‐È.H., G.B. and C.V. Project administration: M.‐È.H. Supervision and funding acquisition: G.B. and M.R.‐C. All authors have read and agreed to the published version of the manuscript.

**Abbreviations**
AGM: African green monkey
BPIV3: Bovine parainfluenza virus type 3
CPD: Codon pair deoptimisation
CT: Cytoplasmic tail
d: day
ERD: Enhanced respiratory disease
F: Fusion protein
FI: Formalin‐inactivated
GMFR: Geometric mean fold rise
HAI: Hemagglutination‐inhibiting
HMPV: Human metapneumovirus
HN: Hemagglutinin‐neuraminidase
HPIV: Human parainfluenza virus
HPIV1: Human parainfluenza virus type 1
HPIV2: Human parainfluenza virus type 2
HPIV3: Human parainfluenza virus type 3
HPIV4: Human parainfluenza virus type 4
IM: Intramuscular
IN: Intranasal
ISG: Interferon‐stimulated gene
IT: Intratracheal
LAV: Live‐attenuated virus
LNP: Lipid nanoparticle
LOD: Limit of detection
LRT: Lower respiratory tract
LRTI: Lower respiratory tract illnesses
mo: month
NA: Not applicable
NAb: neutralising antibody
NDV: Newcastle disease virus
NHP: Non‐human primates
OWL: Old World monkey
postF: Post‐fusion F protein
preF: Pre‐fusion F protein
RM: Rhesus monkey
S: Spike protein
SeV: Sendai virus
SM: Squirrel monkey
SN: Seronegative
SP: Seropositive
TM: Transmembrane
UNS: Unscreened
URT: Upper respiratory tract
URTI: Upper respiratory tract illnesses
yo: year old
